# Supplementary material for: Tumor-intrinsic response to IFNγ shapes the tumor microenvironment and anti–PD-1 response in NSCLC
Source: Life Sci Alliance. 2019 May 27;2(3):e201900328. doi: 10.26508/lsa.201900328 (PMC6537751; doi:10.26508/lsa.201900328)

Western (test) w/ new -correct protein dilutions from Bradford. For socs1 manuscript. 11-16-18.

---

pSTAT1 1:750. same gel as B act. 15ug of protein (91, 84 kD)

Exposure Times

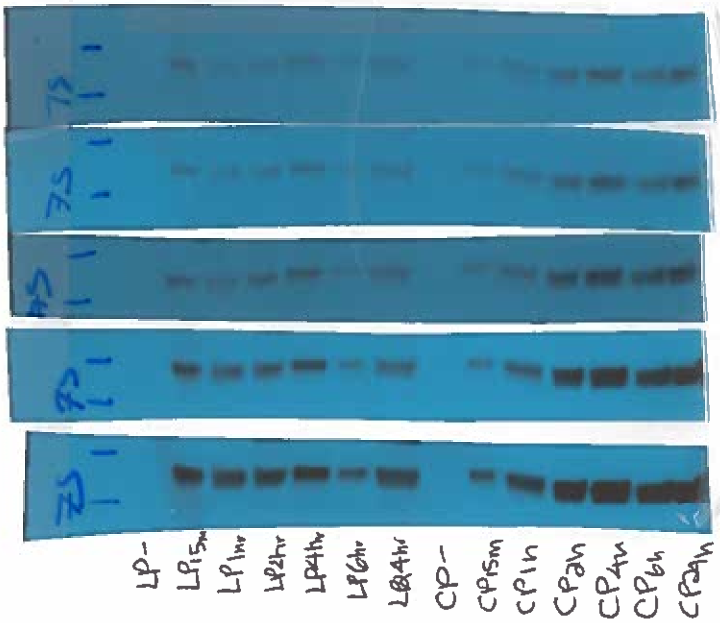

10sec

17sec

20sec ☆.

30sec

1 minute

+/- IFNγ 10ng/ml

B-ACTIN 1:10,000. 15 ug of protein. (42 kD.)

Exposure Times

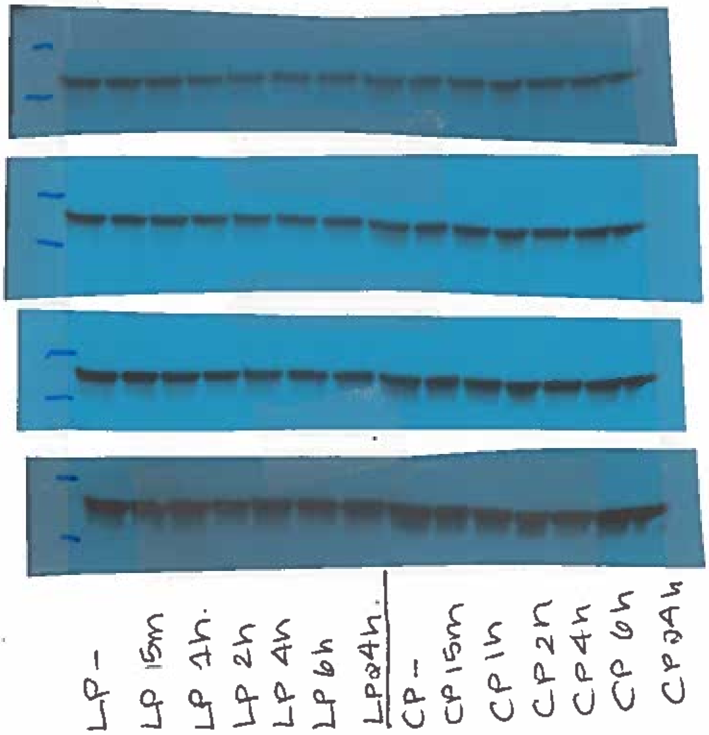

5 sec

10sec ☆.

20sec

30sec.

Pretty equal loading.

Samples. LP- LP 15m LP 1h. LP 2h LP 4h LP 6h LP24h. CP- CP 15m CP 1h CP 2h CP 4h CP 6h CP24h

+/-IFNγ 10ng/ml.

Western: w/correct protein dilutions For SOCS1 Manuscript

11-20-18

Stat1: 1:1500 15ug of protein (91, 84 kD)

Exposure Times

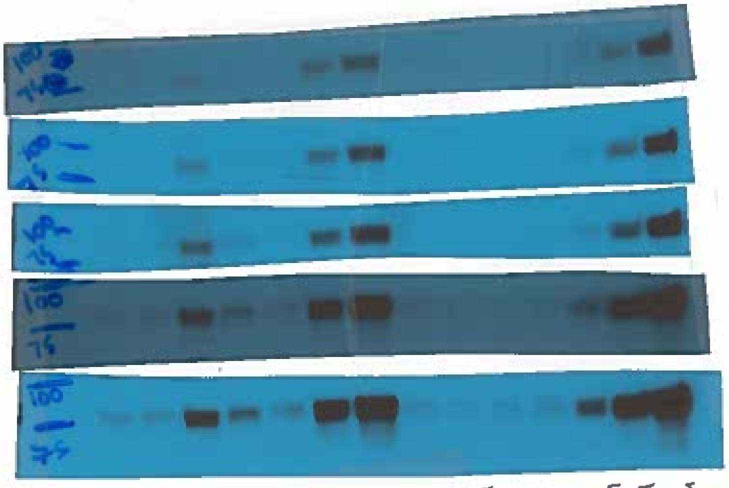

LP- LP15m LP1h LP2h LP4h LP6h LP24h CP- CP15m CP1h CP2h CP4h CP6h CP24h

15 sec

30 sec

1 min

2 min

3 min

+/- IFNγ 10 ng/ml

Corresponding Beta Actin on the Ifngr2 sheet.

These were run w/ same gel.

Western: correct protein dilutions
For Socs1 Manuscript

11-20-18.

Ifngr Beta 1:500 15ug protein = same gel as Stat 1. only can fit ~13 lanes on this blot.
(IFNGR2) * maybe ↑ dil. next time or add more protein.

(49 kD)

Exposure Times
2 min.
7 min. *

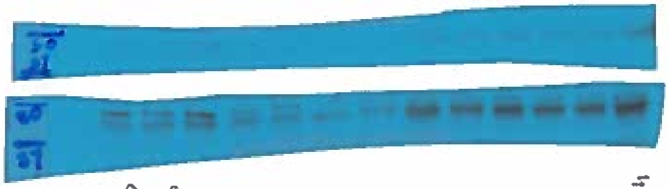

+/- IFNγ 10ng/ml.

β-Actin 1:7500 15ug protein

(42 kD)

Exposure Times
30 sec

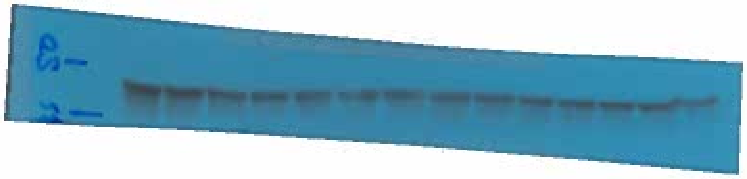

5 sec.

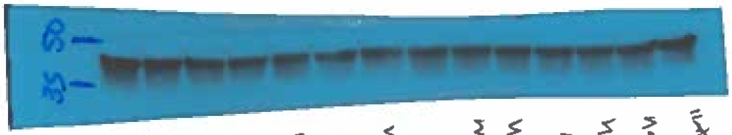

Supplement: Supplementary file 4 [file LSA-2019-00328_SDataF2.pdf]
