# Supplementary material for: Tumor-intrinsic response to IFNγ shapes the tumor microenvironment and anti–PD-1 response in NSCLC
Source: Life Sci Alliance. 2019 May 27;2(3):e201900328. doi: 10.26508/lsa.201900328 (PMC6537751; doi:10.26508/lsa.201900328)
Supplement: Supplementary file 5 [file LSA-2019-00328_SDataFS2.pdf]

Western: Ifngr1 KDs

for Socs1 Manuscript

12/14/18

Ifngr1: LS Bio - CD119/IFNGR1 LS-C334260.

1:1,000 - Runs above 50kD.

Exposure Times

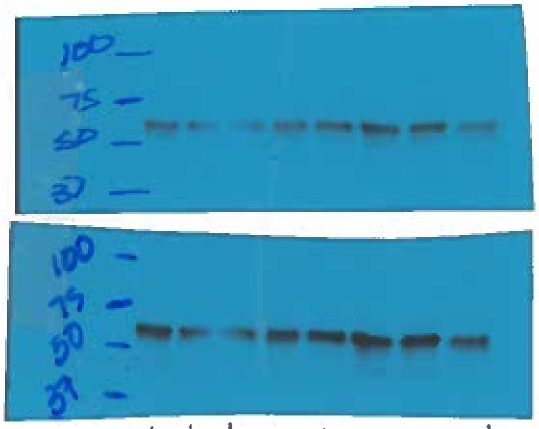

3 sec.

☆ Looks as if theres a KD → but theres potentially a bubble - will rerun.

10 sec.

CP- CN- CG- CS- CP15 LP- LP15 C72-

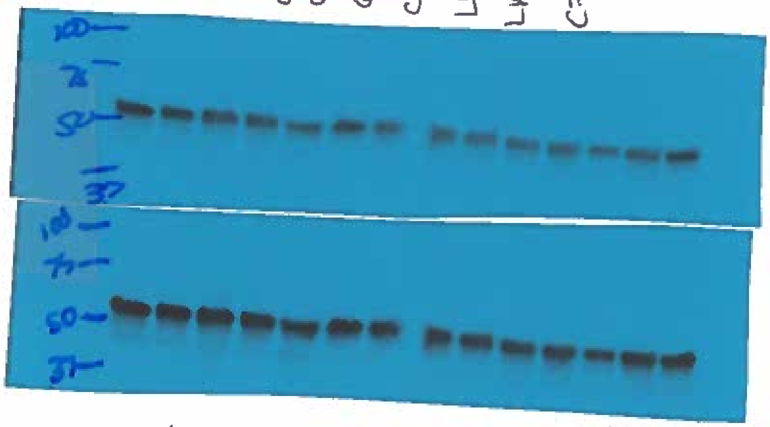

3 sec

☆ Maybe a bubble in the middle?

☆ may also rerun w/ newly diluted samples as a comparison

10 sec.

LP- LP15 LP1 LP2 LP4 LP6 LP24 CP- CP1C CP1 CP2 CP[illegible] CP6 CP24

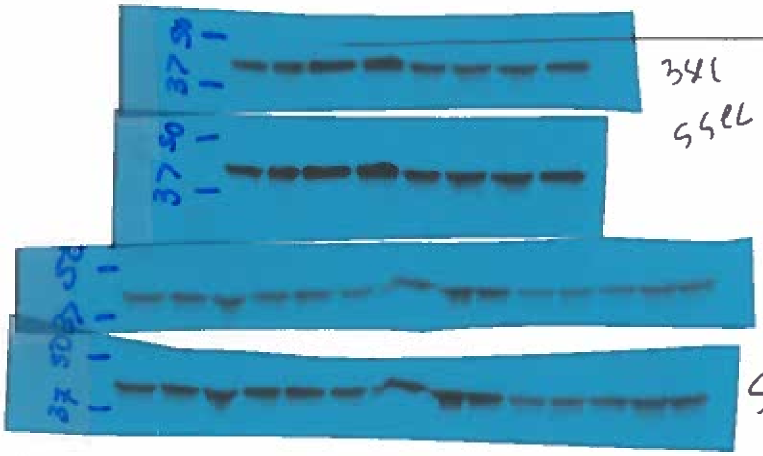

3sec

5sec

Running @ the correct molec weight? ~54 kD.

B Actin

5 sec

3sec

Def. have bubbles. will rerun.

5sec.

5 sec.

Try #2 on other side - not much of a change

Western: correct protein dilutions
For Socs1 Manuscript

11-20-18.

Ifngr2 1:500 15ug protein
Beta = same gel as Stat1. only can't fit ~13 lanes on this blot.
(IFNGR2) ☆ maybe ↑ dil. next time or add more protein.

(49kD)

Exposure Times
2 min.
7 min. ☆.

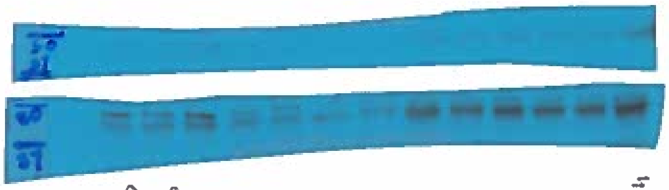

LP- LP15m LP1h LP2h LP4h LP6h LP24h CP- CP15m CP1h CP2h CP4h CP6h CP24h

+/- IFNγ 10ng/ml.

β-Actin 1:7500 15ug protei

(42 kD)

Exposure Times
30 sec

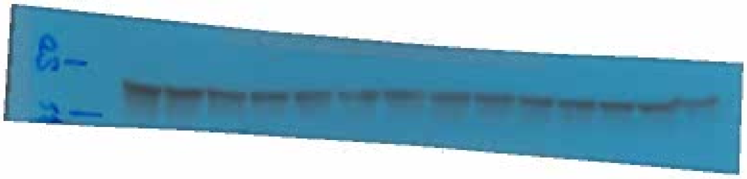

5 sec.

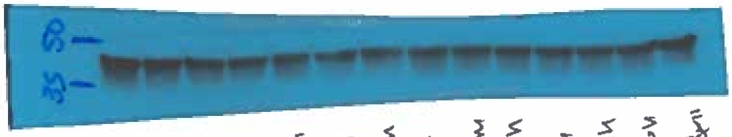

LP- LP15m LP1h LP2h LP4h LP6h LP24h CP- CP15m CP1h CP2h CP4h CP6h CP24h
