# Supplementary material for: Tumor-intrinsic response to IFNγ shapes the tumor microenvironment and anti–PD-1 response in NSCLC
Source: Life Sci Alliance. 2019 May 27;2(3):e201900328. doi: 10.26508/lsa.201900328 (PMC6537751; doi:10.26508/lsa.201900328)
Supplement: Supplementary file 6 [file LSA-2019-00328_SDataF3.pdf]

pSTAT1 Expression

07/17.

CMT-lucs Ifngr1 KDs.
+/- IFN-γ 1hr. 15min. untreated.

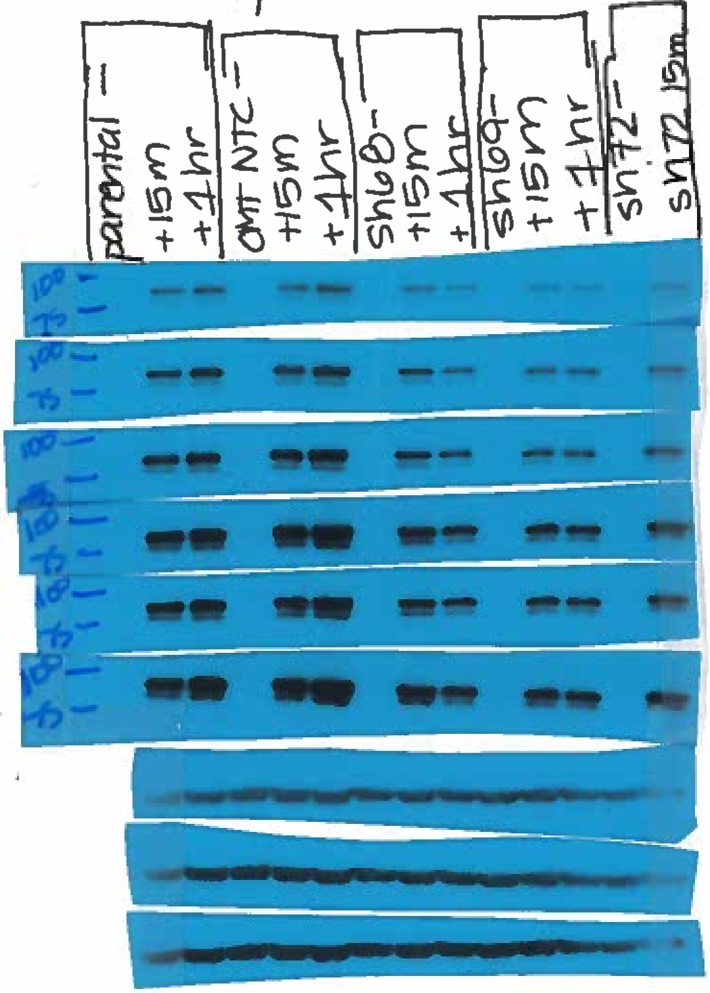

Exposures: pSTAT1
2sec
4 sec
6 sec
8 sec
12 sec
18 sec
Exposures: B.actin
↳ something weird.

10µg of protein loaded:

Best decrease of pSTAT1 expression is seen in the sh69 in all exposures, as compared to parental CMT-lucs and the CMT NTC (non-targeting control cells.

STAT1 Expression

07/17-

CMT-lucs Ifngr1 KDs

+/- IFN-γ 1hr, 15min, untreated.

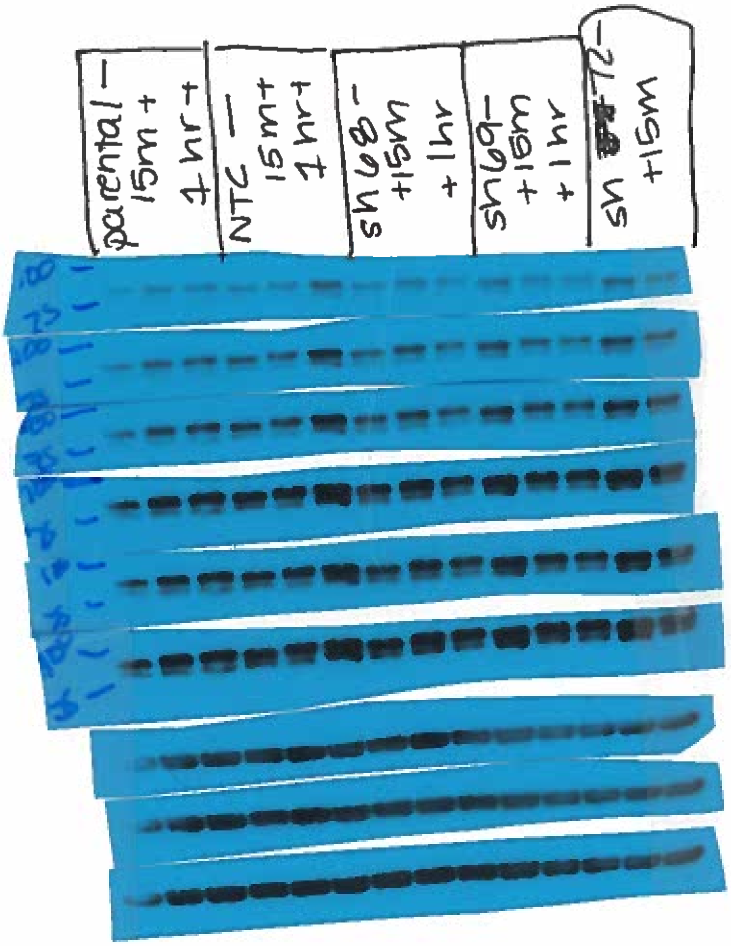

Exposures: STAT1

2 sec

3sec

4sec

8sec

12sec

16sec.

Exposures: β-actin

10ug of protein loaded:

Seems to be a ↓ in stat1 signaling by 1h in the KDs. (sh68/sh69) but not remarkable differences
