# Supplementary material for: Tumor-intrinsic response to IFNγ shapes the tumor microenvironment and anti–PD-1 response in NSCLC
Source: Life Sci Alliance. 2019 May 27;2(3):e201900328. doi: 10.26508/lsa.201900328 (PMC6537751; doi:10.26508/lsa.201900328)
Supplement: Supplementary file 7 [file LSA-2019-00328_SDataF4.pdf]

Western: correct protein dilutions For Socs1 Manuscript

11-20-18

Socs1 : 1:500 15 ug of protein (37 KD)
Maybe ↑ dil. or add more protein next time.

Exposure Times.

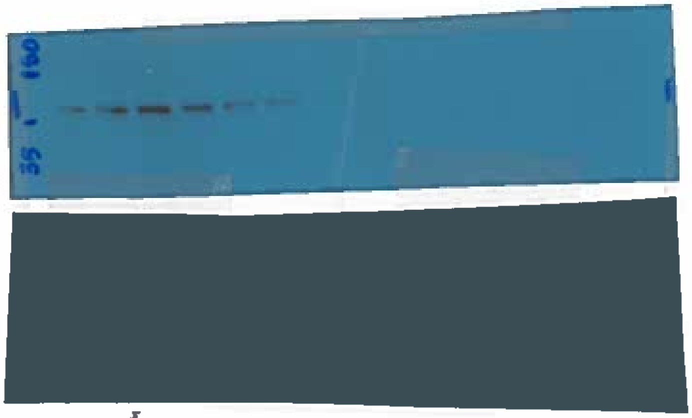

2 minutes.

7 minutes ☆.

LP- LP15m LP1h LP2h LP4h LP6h LP24h CP- CP15m CP1h CP2h CP4h CP6h CP24h

+/- IFNγ 10ng/ml.

B-Actin: 1:7500 15ug protein (42 KD.)

Exposure times

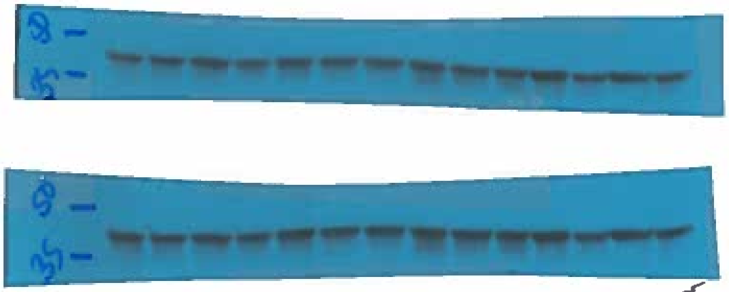

5 sec

10 sec

LP- LP15m LP1h LP2h LP4h LP6h LP24 CP- CP15m CP1h CP2h CP4h CP6h CP24h

Possible socs1 induction w/ IFNγ is regulated by something else by protein in CMTs → or happens later.

LLC-luc Socs1 KD Cells - 1/20/17

Verifying KD. By Protein

Socs1 Ab - Abcam. 1:400.
B-actin Ab - Sigma 1:10,000

−IFNγ: LLC luc par., EV, sh19, sh20, sh21, X
+48hr IFNγ: par., EV, sh19, sh20, sh21, CMT-luc par., CMT-luc par.

Used for pub.

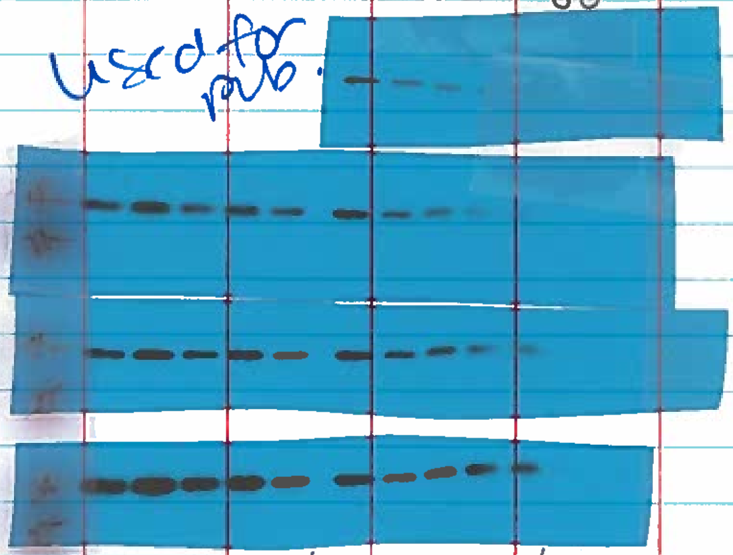

8 sec
20sec.
40sec.
1 min

−IFNγ +48hr IFNγ.

Socs1
✱ Diff. exposure

predicted size is ~24 kD. but certain abs show it running betwe[en] 50-37 kD.

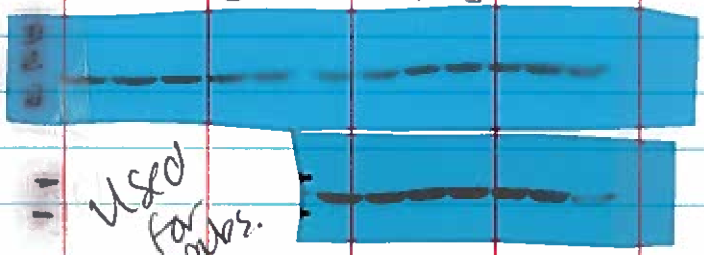

Touch
3sec.

Used for pubs.

B-actin
Diff. exposure. ~42 kD.

Looks like sh19 is a KD compared to EV and the LLC-luc parental line. and possibly sh21.

-should I single cell clone sh19?

TOPS FORM 3519 MADE IN U.S.A.

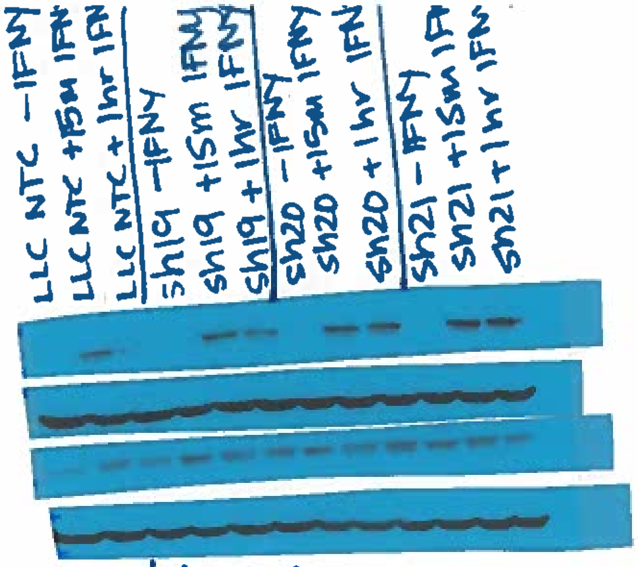

pSTAT1

B-actin for pSTAT1.

STAT1

B-actin for STAT1.

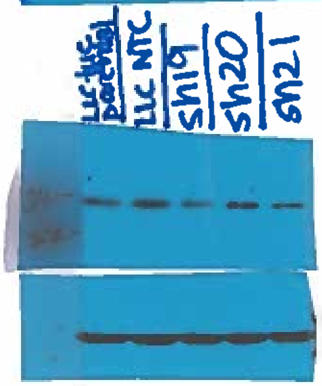

SOCS1 (Abcam)

B-actin for SOCS1.

all −IFNγ.

using for 05/17 SOCS1 publication!
