# Supplementary material for: Tumor-intrinsic response to IFNγ shapes the tumor microenvironment and anti–PD-1 response in NSCLC
Source: Life Sci Alliance. 2019 May 27;2(3):e201900328. doi: 10.26508/lsa.201900328 (PMC6537751; doi:10.26508/lsa.201900328)
Supplement: Supplementary file 8 [file LSA-2019-00328_SDataFS3.pdf]

pSTAT1 - LLC-lucs w/+/- IFNγ | 3.3.17 - 3.5.17

cell lines. older socs1 kD, pools (revalidating)

LEV - | LEV 15m + | LEV 48h + | L19 - | L19 15m + | L19 48h + | L20 - | L20 15m + | L20 48h + | L21 - | L21 15m + | L21 48h + | CMT-luc - | CMT-luc 48h +

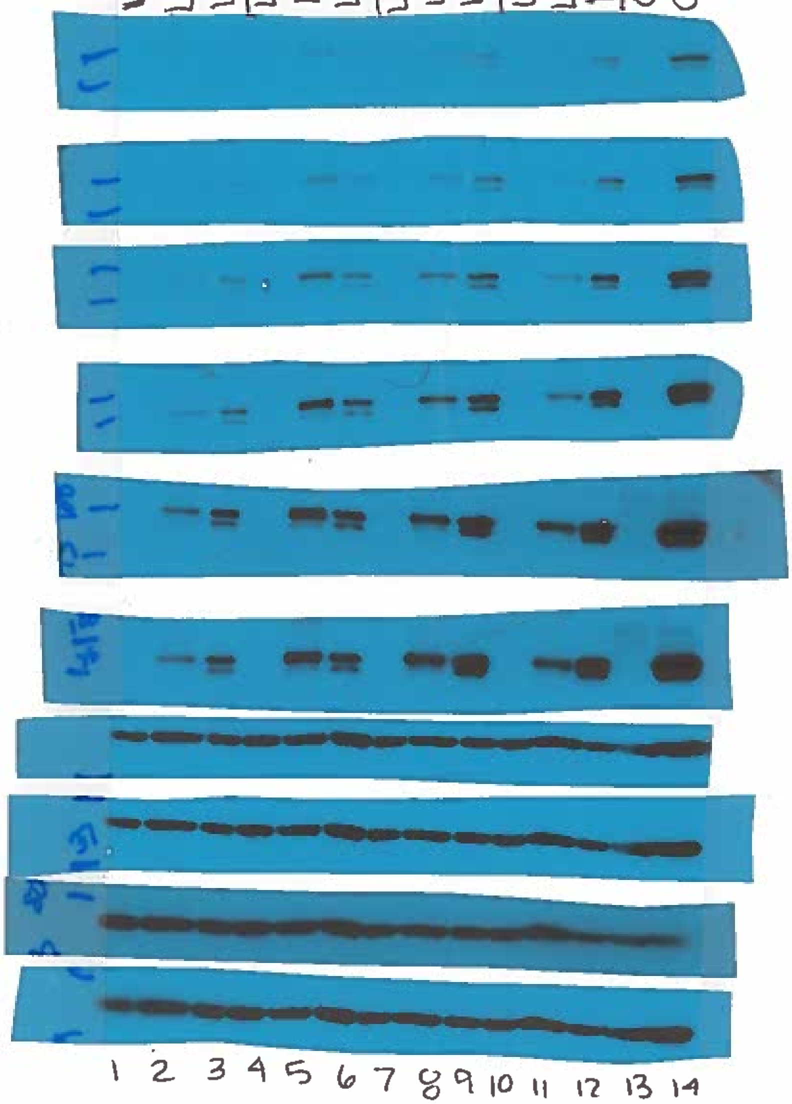

1 2 3 4 5 6 7 8 9 10 11 12 13 14

pSTAT1 Exposures.

3 sec. 1:1000.

5 sec

7 sec

10 sec

30 sec

1 minute.

touch B. actin Exposu[illegible] 1:10,000.

1 sec

2 sec

3 sec.

The EVs induce pSTAT1 at 15m (1 isoform phosphorylated) while @ 48h both isoforms are phosphorylated. sh19, sh20 and sh21 all induce pSTAT1 at 15m and at 48h more robustly than EV ctrl and to a more similar degree to CMTs w/ 48h of IFNγ treatment. This indicates there is a greater magnitude of IFNγ signaling as a function of Socs1 kD in LLC-lucs. Note → these cells were plated @ the same confluency and all treated at the same time. No media was changed, nor IFNγ added more than once.

STAT1 - LLC-lucs w/ +/- IFNγ | 3.3.11 - 3.5.11

cell lines: older SOCS1 KD pools (revalidation)

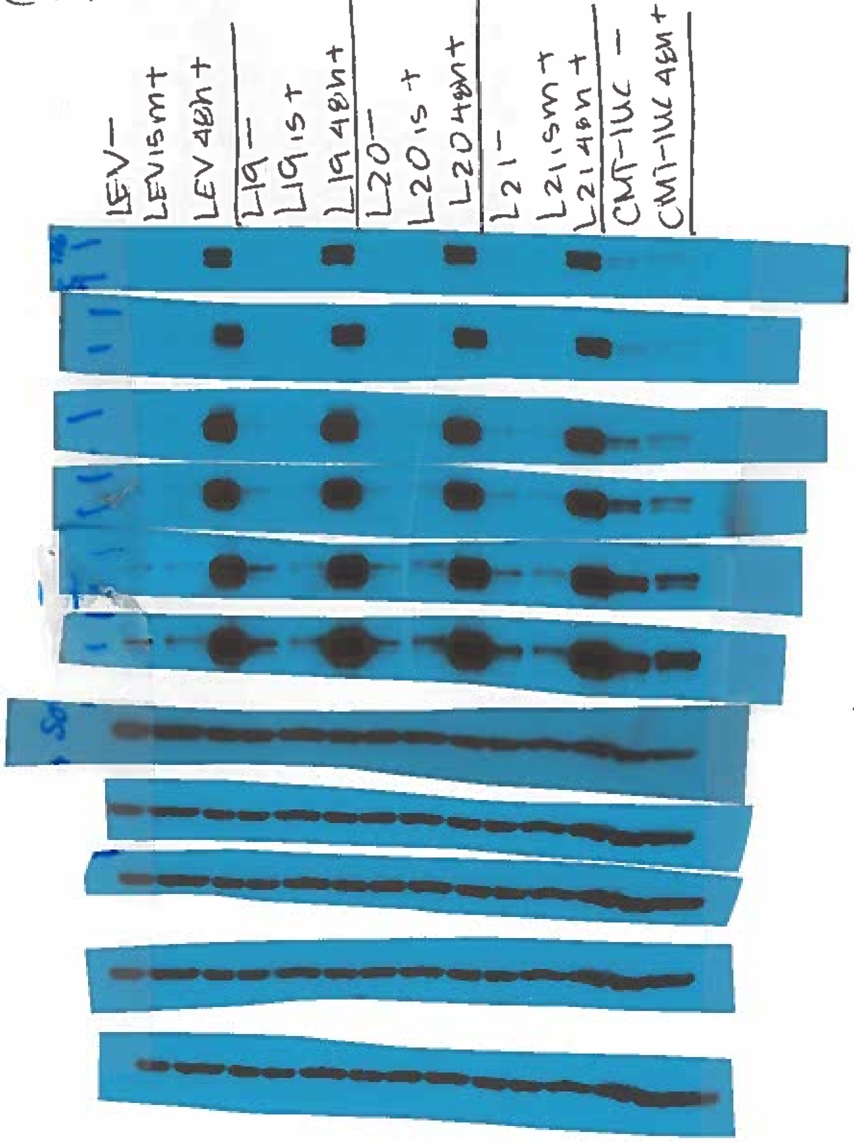

uSTAT1 exposures

→ touch

→ 2 sec

→ 5 sec

→ 10 sec

→ 20 sec

→ 45 sec

1:1,000

B-actin exposure

* all touch.

Interesting that CMTs have high uSTAT1 at both baseline & after 48h IFNγ, but not as much as all the LLC-luc cells. Seems like there are similar uSTAT1 levels by 48h in LLC-luc EV ctrls and KDs. However, it's possible that sh19 and sh21 have high uSTAT1 at baseline compared to EV ctrl. Also looks like there's increasing levels of uSTAT1 in KDs vs EV ctrl by 15 minutes, but less than at baseline perhaps indicates slightly varied transcription programming due to KD of SOCS1.
